# How can Health Behavior Theory be made more useful for intervention research?

**DOI:** 10.1186/1479-5868-1-10

**Published:** 2004-07-23

**Authors:** Robert W Jeffery

**Affiliations:** 1 School of Public Health, Division of Epidemiology, University of Minnesota, Minneapolis, MN 55454-1015, USA

## Abstract

**Background:**

The present paper expresses the author's views about the practical utility of Health Behavior Theory for health behavior intervention research. The views are skeptical and perhaps even a bit exaggerated. They are, however, also based on 20-plus years of in-the-trenches research focused on improving health behavior practice through research.

**Discussion:**

The author's research has been theoretically driven and has involved measurement of varying variables considered to be important theoretical mediators and moderators of health behavior. Regretfully, much of this work has found these variables wanting in basic scientific merit. Health Behavior Theory as we have known it over the last 25 years or so has been dominated by conceptualizations of behavior change processes that highlight cognitive decision-making. Although much of health behavior practice targets what people do rather than what they think, the logic of focusing on thoughts is that what people think about is the key to what they will do in the future, and that interventions that can measure and harness those processes will succeed to a greater extent than those that do not. Unfortunately, in the author's experience, the premise of cognitive theories has fallen short empirically in a number of ways. The cognitive schemata favored by most health behavior theories are difficult to measure, they do not predict behavioral outcomes very well, there is little evidence that they cause behavior, and they are hard to change directly.

**Summary:**

It is suggested that health behavior researchers reconsider their use of these theories in favor of models whose variables are more accessible to observation and experimental manipulation and that most importantly have strong empirical support.

## Background

The author has been conducting research on behavioral treatment of obesity for about 25 years. During that time, the dominant conceptual models guiding intervention development have been cognitive behavior models that have their origin in psychological theory. Those most often cited include the Health Belief Model [1], Protection Motivation Theory [2], Subjective Expected Utility Theory [3], the Theory of Reasoned Action [4], Social Cognitive Theory [5], and the Transtheoretical Model [6]. All of these theories are concerned with how people make behavioral choices and the general idea is that people decide what to do based on the extent to which they expect that their choices will produce results that they value. Much of the content of the theories is concerned with factors that may affect value/expectancy calculations. As summarized by Weinstein in a comparative review of four social psychological theories [7], variables thought to influence value/expectancy judgments include such factors as perceived rewards of current behavior, self-efficacy, normative beliefs, motivation, and the perceived consequences of not changing behavior.

Weinstein's summary is illustrative of the fact that Health Behavior Theory has tended to be particularly interested in understanding people's motivation to change behavior rather than ability to change. Moreover, motivation is thought to be the result of a relatively complex, but logical, interpretation of large quantities of information about self and environment. The theories that Weinstein reviewed deal almost exclusively with behavioral decision processes in people's minds. They have few if any terms relating to how information gets into peoples minds or how subsets of it receive more or less attention. Broader health behavior theories such as Social Cognitive Theory or the Transtheoretical model have addressed issues and variables outside the person to a greater extent, but the fundamental interest in and belief in psychological variables as the key force in determining health behavior remains.

The implications of the focus of health behavior theory on psychological determinants of behavioral decision-making for my own research area of interest, obesity treatment, are several. One is the inclusion of measures of psychological characteristics in most research protocols (e.g., assessment of behavioral intentions, self-efficacy, perception of barriers to change, perception of social support, and outcome expectations). A second is the inclusion of treatment elements that specifically target psychological perceptions and processes independent of the diet and physical activity behaviors that actually produce weight change (e.g., how to deal with emotional eating, how to deal with the frustration of lapses and relapses, and how to talk to yourself to increase self-motivation). A third is the belief that psychological reactions to treatment experiences themselves are very important and deserve independent attention. Common behavioral prescriptions for weight-loss goals and frequency of self-weighing are exemplary (i.e., recommending infrequent weighing to prevent discouraging feedback about progress and encouraging smaller and thus "more attainable" behavior and weight-loss goals in the belief that they will be more motivating).

The problem with the emphasis on cognitive variables in weight-control research is that they have so far failed to meet fundamental scientific criteria for empirical verification. Thus, they also have not led to a better understanding of the weight-loss process, have not improved our ability to predict weight-loss outcomes, and have not led to improvement in treatment methods. In some cases it is even arguable that they have made treatment worse. I will illustrate these problems with results from my own research.

## Discussion

Like most behavioral researchers in the obesity area, I have attempted to measure elements of health behavior theory in every obesity intervention project I have ever conducted. I have assessed weight-loss goals, behavioral and weight-loss self-efficacy, psychological well-being, perceived barriers to diet and physical activity change, stages-of-change, and perceived social support. How well have empirical examinations of these factors fared as predictors of success in weight control?

### Self-efficacy

We have examined the predictive value of self-efficacy assessments in several of our studies and describe the results from three of these here in more detail [8-10]. In the first study, self-efficacy was assessed at baseline, posttreatment, and one year later in 85 men participating in a 15-week weight-loss program [8]. The self-efficacy instrument had subscales for emotional states (e.g., anxiety) and situations (e.g., eating away from home). Higher baseline self-efficacy on both subscales was associated with greater weight loss in treatment and at 1- and 2-year follow-up. Emotional self-efficacy at posttreatment did not predict weight loss at 1- or 2-year follow-up. Situational self-efficacy at posttreatment predicted weight loss at 1-year but not 2-year follow-up.

The second study examined mood and situational self-efficacy in 55 men and 58 women before and after a 16-week weight-loss treatment with a 1-year follow-up [9]. Women had lower pretreatment self-efficacy than men. Self-efficacy was predictive of weight loss and maintenance in men but not in women. Change in self-efficacy over time was positively related to weight change in women but not in men.

The third study examined predictors of weight change over a 2-year period in 460 men and 1172 women who received a low-intensity weight-loss intervention delivered through their HMO [10]. The self-efficacy measure was the WEL questionnaire. Men again were found to have higher baseline self-efficacy than women. Self-efficacy did not predict weight change in men but was positively, though weakly, related to weight change at 6 months only in women.

Our overall conclusion from the analyses described above, as well as others not pursued in as great detail, is that self-efficacy is a weak predictor of weight loss and is inconsistent across study populations and gender. It tends to increase with weight loss. However, treatment-induced increases in efficacy are not predictive of longer-term weight-loss success.

### Barriers to Adherence

We have also attempted to measure barriers to adherence to weight-control behaviors in many of our studies [11-14]. The instruments used for this have typically been formatted similarly to efficacy questionnaires in that people are asked to indicate how difficult they find situational, knowledge, and motivational challenges to achieving diet and exercise changes. The findings in these studies have been quite consistent. Baseline assessments of perceived barriers to behavior change are not predictive of weight change. Weight loss is associated with reported decreases in perceived barriers. Treatment-induced change in perceived barriers are not predictive of future weight change. In other words, barrier perceptions as we have measured them do not appear to have pragmatic significance.

### Weight Goals

Goal-setting has long been of interest to health behavior theory and in recent years has attracted attention in weight-loss research when it was realized that most people who enter weight-loss treatments want to lose a lot more weight than is realistic given the potency of current weight-loss methodologies [15]. When asked to describe weight losses they deem to represent "dream, happy, acceptable, and disappointing," many individuals in treatment fail to reach even "disappointing" weight losses even though in objective medical terms the results are positive. Based on the argument that failure to reach gratifying weight-loss goals leads to psychological distress that lowers weight self-efficacy and undermines weight-loss efforts, it has become popular to recommend counseling in weight-loss treatments specifically targeting the lowering of weight-loss goals. The theoretical argument is that excessive outcome expectations undermine behavioral efforts. We have now completed three sets of formal analyses examining whether weight goals are predictive of weight-loss success. In one of these analyses the relationship between weight-loss goals, weight-loss goal attainment, and long-term (30 months) weight-loss attainment and psychological well-being were assessed in 69 men and 61 women participating in an intensive behavioral treatment program [16]. Results indicated that weight-loss goals were unrealistically high on average and that lower goals were more likely to be reached. Nevertheless, weight-loss goals did not predict either short- or long-term weight losses and were not associated with elevated psychological distress. Two more recent analyses we have conducted looking at weight-loss goals as predictors of success have produced similar results [Linde JA, Jeffery RW, Levy RL, Pronk NP and Boyle RG, unpublished data [17]]. Weight-loss goals either did not predict weight loss at all or were slightly positively related to weight-loss success.

### Perceived Social Support

Perceived social support is another psychological factor thought to influence health behavior decision-making. We have measured social support in a variety of ways in our studies, ranging from single-item questions to multipaged assessments attempting to differentiate among informational, instrumental, and emotional support. The results, unfortunately, have closely paralleled those we have seen with other assessments of barriers to adherence. Assessments of social support prior to treatment do not predict weight loss. Average reports of social support tend to parallel weight loss itself. When people lose weight they report more social support. When they regain, they report less. In other words, perceptions of social support are not predictive of success in weight-loss treatments.

### Frequency Weight Self-monitoring

Self-monitoring of health behavior is incorporated into many health behavior theories, usually as part of a person's assessment of achieved outcomes. Although self-monitoring is usually considered a positive element in the adoption of health behavior, in obesity treatment frequent self-monitoring of weight has tended to be down-played or even discouraged on the grounds that disappointing results (i.e., less than desired weight change) may undermine motivation. This is another example in which health behavior theory may have indirectly led to incorrect treatment recommendations. In weight-loss treatments, active discouragement of frequent self-observation of weight has become popular based on the premise that more frequent weighting will cause psychological stress and lower self-efficacy. Recently, we have examined the relationship between frequency of self-weighing and body weight in both clinical and population samples and have found, somewhat to our surprise, that frequency of self-weighing is one of the strongest single predictors of body weight cross-sectionally, and change in the frequency of self-weighing is one of the strongest predictors of weight change [Linde JA, Jeffery RW and French SA, unpublished data]. The direction of predictions, however, is opposite that derived from theory. People who weigh themselves more weigh less and are more successful in losing weight.

### Stage-of-Change

A final failure of current health behavior theory to prove useful in weight-control research is a recent examination of the relationship between a stage-of-change measure adopted from Prochaska and short- and long-term weight loss [18]. Categories of precontemplation, contemplation, preparation, and action were defined based on questions about weight-loss intentions and recent weight-loss attempts. Despite a large sample size, excellent follow-up rates, and well-measured objective outcomes, we were unable to demonstrate that staging algorithms recommended by proponents of the Transtheoretical Model could predict weight-loss outcomes.

### Experimental Modification of Expectations

Our most recent effort to utilize health behavior theory in obesity intervention research is a study that attempted to examine the effectiveness of experimentally-induced outcome expectancies on weight loss [Finch EA, Linde JA, Jeffery RW, Rothman AJ and King CM, unpublished data]. Obese men and women participated in an 8-week weight-loss program with 18-month follow-up in which they were assigned to one of two expectancy groups. The optimistic group was told that focusing exclusively on the positive benefits of weight loss would be valuable in ensuring that they remained motivated in their weight-loss efforts and was given assignments during weekly group sessions and homework between sessions to reinforce this optimistic mindset. A "balanced" expectancy group received the instructions that focusing on both the positive and negative aspects of weight loss, a balanced approach, would be most conducive to maintaining weight-loss motivation. This group also received assignments to reinforce their message. Results of this study indicated that the expectation induction was successful initially but difficult to maintain in the face of real weight-loss experience. We were also unable to show that experimentally-induced expectations influenced weight-loss success.

## Summary and Conclusion

To summarize the findings described above, I have had considerable difficulty over the last 25 years in confirming that the psychosocial variables favored by health behavior theory are of much value for obesity intervention research. They do not predict weight loss well, either as mediators or moderators. There is little evidence to support the idea that targeting them for intervention improves weight-loss outcomes. It is, of course, arguable that the weak findings relating to health behavior theory variables are due in large part to methodological weaknesses, either in measurement tools and/or their frequency of measurement. I would argue, however, that 25 years is long enough to wait for improved methods and that it is time to look elsewhere for variables that better predict weight-change outcomes and that, therefore, may form a better basis for improving future treatments.

### Implication for Weight-Loss Treatment

Given the lack of success finding support for cognitive mediators of behavior change in weight loss, one might surmise that progress in improving weight-loss interventions over the last 20 years must have been dreary indeed. Somewhat surprisingly, however, that is not the case. In fact, the short-term (6 to 12 months) success of weight-loss treatments has approximately doubled over that time and several variables have been identified that reliably enhance treatment outcomes. It has been clearly shown experimentally that increasing treatment length [19], prescribing low-energy intakes [20], prescribing high-energy expenditure [21], using a deposit contract and group-based reward systems [22], and simplifying adherence to diet through meal substitutes [23] and exercise by providing exercise equipment [24] all improve initial weight loss. From a theoretical perspective, however, one thing is noteworthy about these successful innovations. Although not incompatible with health behavior theory, none of them are specifically derived from cognitive decision-making models. Indeed, health behavior theory does not include variables like these in its models.

### Where Do We Go From Here?

The argument above about the practical limitations of many popular theories of health behavior is not meant to be a call to abandon theory. Behavior scientists have amassed much useful information about the principles underlying human behavior that should be valuable for health behavior interventions. Much is known about human perception, learning, motivation, and responsiveness to environmental opportunities and contingencies. Health behavior intervention lies at the interface between people and their environment. Interventionists change aspects of the environment (cues, information, behavioral contingencies) with the intention of producing changes in how people behave. What is needed to advance health behavior intervention is theory that addresses relationships between modifiable aspects of the environment and behavior. There is no doubt that cognitive processes are involved in these relationships. However, the extent to which current theories capture this is questionable. Data now available suggest that easily obtainable information about people's cognitive processes adds little to our ability to predict the results of interventions. Thus, it may be wise to pay more attention to applied theories like classical behavior theory [25], communications theory [26], and learning theory [27] than to those coming out of the social cognitive traditions.

## Competing interests

None declared.

## References

[B1] Becker MH, Ed (1974). The health belief model and personal health behavior. Health Education Monographs.

[B2] Maddux JE, Rogers RW (1983). Protection motivation and self-efficacy: a revised theory of fear appeals and attitude change. J Exp Soc Psychol.

[B3] Edwards W (1954). The theory of decision making. Psychol Bull.

[B4] Ajzen I, Fishbein M (1980). Understanding Attitudes and Predicting Behavior.

[B5] Bandura A (1986). Social Foundations of Thought and Action: A Social Cognitive Theory.

[B6] Prochaska JO, Velicer WF (1997). The transtheoretical model of health behavior change. Am J Health Promot.

[B7] Weinstein ND (1993). Testing four competing theories of health-protective behavior. Health Psychol.

[B8] Jeffery RW, Bjornson-Benson WM, Rosenthal BS, Lindquist RA, Kurth CL, Johnson SL (1984). Correlates of weight loss and its maintenance over two years of follow-up among middle-aged men. Prev Med.

[B9] Forster JL, Jeffery RW (1986). Gender differences related to weight history, eating patterns, efficacy expectations, self-esteem, and weight loss among participants in a weight reduction program. Addict Behav.

[B10] Linde JA, Jeffery RW, Levy RL, Sherwood NE, Utter J, Pronk NP, Boyle RG (2004). Binge eating disorder, weight control self-efficacy, and depression in overweight men and women. Int J Obes Metab Discord.

[B11] Jeffery RW, Wing RR, Thorson C, Burton LR, Raether C, Harvey J, Mullen M (1993). Strengthening behavioral interventions for weight loss: A randomized trial of food provision and monetary incentives. J Consult Clin Psychol.

[B12] Wing RR, Jeffery RW, Burton LR, Thorson C, Sperber Nissinoff K, Baxter JE (1996). Food provision vs structured meal plans in the behavioral treatment of obesity. Int J Obes Relat Metab Discord.

[B13] Jeffery RW, Wing RR, Thorson C, Burton LR (1998). Use of personal trainers and financial incentives to increase exercise in a behavioral weight-loss program. J Consult Clin Psychol.

[B14] Jeffery RW, Wing RR, Sherwood NE, Tate DF (2003). Physical activity and weight loss: does prescribing higher physical activity goals improve outcome?. Am J Clin Nutr.

[B15] Foster GD, Wadden TA, Vogt RA, Brewer G (1997). What is a reasonble weight loss? Patients' expectations and evaluations of obesity treatment outcomes. J Consult Clin Psychol.

[B16] Jeffery RW, Wing RR, Mayer RR (1998). Are smaller weight losses or more achievable weight loss goals better in the long term for obese patients?. J Consult Clin Psychol.

[B17] Linde JA, Jeffery RW, Finch EA, Ng DM, Rothamn AJ (2004). Are unrealistic weight loss goals associated with outcomes for overweight women?. Obes Res.

[B18] Jeffery RW, French SA, Rothman AJ (1999). Stage of change as a predictor of success in weight control in adult women. Health Psychol.

[B19] Perri MG, Nezu AM, Patti ET, McCann KL (1989). Effect of length of treatment on weight loss. J Consult Clin Psychol.

[B20] Wadden T, Foster G, Letizia KA (1994). One-year behavioral treatment of obesity: comparison of moderate and severe calorie restriction and the effects of weight maintenance therapy. J Consult Clin Psychol.

[B21] Pronk NP, Wing RR (1994). Physical activity and long-term maintenance of weight loss. Obes Res.

[B22] Jeffery RW, Hirsch J, Van Itallie TB (1983). Monetary contracts for weight loss. In Recent Advances in Obesity Research: IV (Proceedings of the 4th International Congress on Obesity, New York, 1983).

[B23] Wing RR, Jeffery RW (2001). Food provision as a strategy to promote weight loss. Obes Res.

[B24] Jakicic JM, Wing RR, Butler BA, Jeffery RW (1997). The relationship between presence of exercise equipment in the home and physical activity level. Am J Health Promo.

[B25] Rachlin H, Atkinson RC, Lindzey G, Thompson RF (1935). Judgment, Decision, and Choice: A Cognitive/Behavioral Synthesis In A Series of Books in Psychology.

[B26] Lefebvre RC, Flora JA (1988). Social marketing and public health intervention. Health Educ Q.

[B27] Baddeley A (1990). Human Memory: Theory and Practice.

